# Morpho-Anatomical Traits and Soluble Sugar Concentration Largely Explain the Responses of Three Deciduous Tree Species to Progressive Water Stress

**DOI:** 10.3389/fpls.2021.738301

**Published:** 2021-12-07

**Authors:** Jonathan O. Hernandez, Ji Young An, Marilyn S. Combalicer, Jong-Pil Chun, Sang-Keun Oh, Byung Bae Park

**Affiliations:** ^1^Department of Environment and Forest Resources, Chungnam National University, Daejeon, South Korea; ^2^Department of Forest Biological Sciences, College of Forestry and Natural Resources, University of the Philippines Los Baños, Los Baños, Philippines; ^3^Institute of Agricultural Science, College of Agriculture and Life Sciences, Chungnam National University, Daejeon, South Korea; ^4^Department of Horticulture, Chungnam National University, Daejeon, South Korea; ^5^Department of Applied Biology, Chungnam National University, Daejeon, South Korea

**Keywords:** Adaptive mechanisms, *Betula schmidtii*, drought-stress, drought response, tolerance, tree physiology, *Quecus acutissima*, *Quercus serrata*

## Abstract

A better understanding of plant drought responses is essential to improve plant water use efficiency, productivity, and resilience to ever-changing climatic conditions. Here, we investigated the growth, morpho-anatomical, physiological, and biochemical responses of *Quercus acutissima* Carruth., *Quercus serrata* Murray, and *Betula schmidtii* Regel to progressive water-stress. Seedlings were subjected to well-watered (WW) and water-stressed (WS) conditions while regularly monitoring the soil volumetric water content, stem diameter (SD), height, biomass, stomatal conductance (g_s_), intercellular CO_2_ concentration (C_i_), and leaf relative water content (RWC). We also investigated the variation in stomatal pore (SP) area, specific leaf area (SLA), root xylem vessel diameter (VD), and total soluble sugar (TSS) concentration between treatments. After 2 months, WS significantly suppressed SD growth of *Q. acutissima* and *B. schmidtii* but had no impact on *Q. serrata*. Total biomass significantly declined at WS-treated seedlings in all species. WS resulted in a smaller SLA than WW in all species. The SP of WS-treated seedlings of *Q. acutissima* and *B. schmidtii* significantly decreased, whereas it increased significantly with time in *Q. serrata*. Larger vessels (i.e., >100 to ≤ 130) were more frequent at WS for *Q. acutissima* and *B. schmidtii*, whereas smaller vessels (i.e., >40 to ≤ 90) were more frequent at WS than at WW for *Q. serrata* after 8 weeks. Tylosis was more frequent at WS than WW for *Q. serrata* and *B. schmidtii* at eighth week. WS seedlings showed lower g_s_, C_i_, and RWC compared with WW-treated ones in *Q. acutissima* and *B. schmidtii*. TSS concentration was also higher at WS-treated seedlings in two *Quercus* species. Overall, principal component analysis (PCA) showed that SLA and SP are associated with WS seedlings of *Q. serrata* and *B. schmidtii* and the tylosis frequency, TSS, and VD are associated with WS seedlings of *Q. acutissima*. Therefore, water-stressed plants from all species responded positively to water stress with increasing experimental duration and stress intensity, and that is largely explained by morpho-anatomical traits and soluble sugar concentration. The present study should enhance our understanding of drought-induced tree growth and short-term tree-seedling responses to drought.

## Introduction

Droughts are expected to occur more often at larger spatio-temporal scales due to increasing global warming and unprecedented impacts of climate change (Han and Singh, 2020). Approximately 80%–90% of the decline in the global gross primary production are caused by drought events through widespread tree mortality and rapid decline in forest carbon uptake and sequestration (Zscheischler et al., 2014; Buckley, 2019; Sharma and Zheng, 2019; Pennisi, 2020). Depending on the intensity and duration, drought stress can result in severe disorders in plant functions, and then eventually intensify tree mortality and alterations in the structure and compositions of forest ecosystems (Allen et al., 2010; Berg and Sheffield, 2018; Kapoor et al., 2020). Plant drought responses generally include reduced rates of stomatal conductance and photosynthesis, which negatively affect the carbon exchange between vegetation and atmosphere (Farooq et al., 2009; Deka et al., 2018; Bangar et al., 2019). Thus, understanding the mechanisms or resistance strategies exhibited by the water-stressed plants can help us to explain the plant resilience amid frequent drought episodes caused by climate change.

Many studies have already reported different effects of water stress on plant growth, morpho-anatomy, physiology, and biochemistry. For example, leaf wilting, reduced height, root collar diameter, and total biomass and altered root and shoot phenology are some of the reported effects of plants exposed to severe water stress (Bhatt and Rao, 2005; Razmjoo et al., 2008). In some plants, water deficit conditions improved root to shoot proportion or root biomass allocation as an important strategy of plants to enhance the water balance (Braatne et al., 1992; Tschaplinski et al., 1994). Water stress can cause alterations in a range of leaf morpho-anatomical characteristics, such as in leaf thickness, xylem vessels, occlusions, leaf biomass, leaf area, and specific leaf area (SLA) (Bhargavi et al., 2017; Byambadorj et al., 2021). Some studies reported that a reduction in leaf area of drought-exposed plants is a mechanism to maintain cell turgidity and achieve stability among the water absorbed by the roots (Najla et al., 2012; Srivastava and Srivastava, 2014). An investigation by Haworth et al. (2017) reported that water stress treatment resulted in an increased xylem vessel diameter and movement of water after the experiment, but was more prone to embolism compared with smaller ones (Cochard, 2002; Smith et al., 2013). Physiologically, plant responses to water stress can be first seen in the alterations in cell growth and structure caused by turgor loss and a decrease in stomatal conductance, chlorophyll content, and photosynthesis (Sanchez et al., 1982; Tanaka et al., 2013). These physiological effects occurred primarily due to closing of stomata, decreasing leaf water content and/or water potential, malfunctioning of enzymes, and cellular damage (Kumawat and Sharma, 2018; Sharma et al., 2020). Lastly, plant water status is also affected by the accumulation of solutes, e.g., amino acids, soluble sugars, and other osmolytes, which are involved in the plant metabolism and growth (Falchi et al., 2020). For example, a 2-month drought stress experiment resulted in a significant increase in the amount of soluble sugars in leaves of drought-tolerant *Quercus pubescens* Willd (Holland et al., 2015). The presence or accumulation of soluble sugars has long been considered a mechanism for tolerating water stress in some studies (Wang et al., 1995; Yakushiji et al., 1996; Ogawa and Yamauchi, 2006; Masouleh et al., 2019).

However, the challenge remains to elucidate responses of forest tree species to water stress due to a high degree of inter- and intra-species specificity and unpredictable nature of drought (Gray and Brady, 2016; Seleiman et al., 2021). For example, based on the stress resistance syndrome (SRS), forest tree species may respond to water stress in various ways. Species adapted to low-resource habitats tend to conserve resources (e.g., water) under unfavorable conditions to increase survival at the expense of growth, by lowering photosynthesis rate and resource uptake, whereas species adapted to high-resource environments (e.g., deciduous species) are usually more plastic to resource fluctuations (Aerts and Chapin, 2000). Previous studies have also reported that plant responses to water stress are highly variable depending on the stress duration and intensity, stage of plant development, and complexity of net physiological and morphological changes (Lum et al., 2014; Schneider et al., 2018). A meta-analysis, for example, showed that the negative effects of water stress on chlorophyll were more evident at higher stress intensity and duration (Sun et al., 2020). Moreover, only a few studies have measured multiple traits using different forest tree species under progressive stress to understand different response mechanisms indicative of water stress resistance (Reynolds et al., 2017).

Thus, we investigated the growth, morpho-anatomical, physiological, and biochemical responses of deciduous *Quercus acutissima* Carruth., *Quercus serrata* Murray, and *Betula schmidtii* Regel to progressive water stress and examine the drought response strategies of each species. Although the drought resistance of these species was already recognized in several studies, most of them focused on only one species and did not measure multiple drought resistance traits under the progressive water stress across different tree species. Because deciduous species delay drought stress by shedding leaves and maximize resource acquisition when resources are limited, we hypothesized that water-stressed plants from all species would respond positively to water stress with increasing experimental duration and stress intensity. In particular, we would expect little variation between well-watered (WW) and water-stressed (WS) treatments in all the drought resistance traits measured across time periods. The results of the present study should enhance our understanding of drought-induced tree growth and short-term tree-seedling responses to drought.

## Materials and Methods

### Study Area

This study was conducted in a greenhouse at Chungnam National University (36°22′12″N, 127°21′17″E) located in Yuseong-gu, Daejeon, the Republic of Korea from May to September 2020. The mean daily air temperature and relative humidity were 24.8°C and 78.17%, respectively (Supplementary Figure 1). These environmental data were collected in the greenhouse using the Onset HOBO air temperature sensor (Optic USB Base Station, U23 Pro v2). To reduce the effect of summer temperature, a knitted shade cloth was installed on the roof of the greenhouse and the lower half of the window was kept open, except during rainy days. A pesticide (Etofenprox and Imidacloropid) was also sprayed on the plants to control the spread of destructive insects.

### Plant Materials, and Experimental Design

We used 1-year-old containerized seedlings of *Quercus acutissima* Carruth., *Quercus serrata* Murray, and *Betula schmidtii* Regel with similar initial stem diameter (SD) and height growth. These species are all perennial, deciduous, broadleaf, fast-growing species, occurring in a range of different habitats from low to high elevations (Li et al., 2005; Saito et al., 2017). Two hundred seedlings of each species, which were obtained from well-managed commercial nurseries in Korea, were planted in March 2020 in a 450 L pot, filled with artificial soil (Baroker, Inc., South Korea). The soil used was loamy sand in texture, with 7.01 pH, 7.28 cmol kg^–1^ CEC, and 1.29% soil organic matter. No fertilizer was added to the soil.

The open-bottom pots were organized in a completely randomized experimental design encompassing 10 replicates for each treatment and species. Ten seedlings were planted in each pot, and replacement of inferior ones was done 1 month after planting. Before water stress treatment imposition, seedlings underwent an acclimatization period for 5 months to establish roots and acclimate to the greenhouse environmental conditions. We monitored the root growth, the fine root in particular, using a rhizobox installed in the pot to make sure that all seedlings were all ready to water stress treatment imposition from the beginning until the end of the experiment. During this period, all seedlings were watered every 2 days until treatment imposition.

**FIGURE 1:**
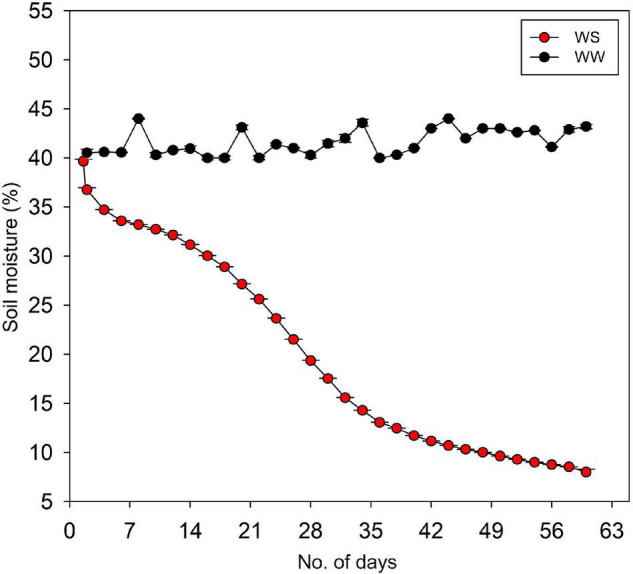
Weekly soil volumetric water content of well-watered (WW) and water-stressed (WS) pots containing the seedlings of *Quercus acutissima*, *Quercus serrata*, and *Betula schmidtii*. Each data point is the average of the daily values which were logged at a 5-min interval.

When seedlings had started to develop secondary roots and height growth of approximately 0. 9–1.2 m, two water stress treatments were imposed: WW (control) and WS seedlings. In the WW treatment, the soil VWC (volumetric water content) was maintained at 40–45%, which is the field capacity of the soil after 2 days. In contrast, the VWC at WS treatment was allowed to decline gradually from 40 to only 8%, which is the permanent wilting point of the soil, until the end of the experiment (Figure 1). The stress imposition was done from the first week of August to the last week of September. Water stress was imposed following a modified method in Jimenez et al. (2013) and the theoretical basis of drought experiment or plant–soil water interactions described in Snow and Tingey (1985).

The VWC was monitored by frequency domain technology (70 MHz, FDT), with 5-cm-length probes (ECH_2_O EC-5, *n* = 4) vertically inserted into the pot. These probes were connected to a data logger (ZL6, Meter Group Inc., United States) to collect the data on-site through ZENTRA utility application. The data from each soil sensor were logged every 5 min throughout the duration of the experiment.

### Growth Measurement

To account for the edge effects, two seedlings in the middle of each pot were selected as the subjects of the measurements. Two weeks before treatment application, we measured the plant height (HT) and stem diameter (SD) weekly until the end of the experiment. On the bark of a seedling, we put a permanent mark (i.e., 1 cm from the soil surface), on which the SD was measured using a digital caliper (in mm). Measurement of the plant HT was done by measuring it from the permanent SD-mark up to the highest terminal bud of an orthotropic branch using a 2-m foldable ruler.

At the end of the experiment, seedlings were harvested for biomass growth determination. Plant components were separated into leaf, stem, branch, and root. Their respective biomasses and the total biomass were then determined using the oven-drying method. Samples were weighed at 65°C for 48 h.

### Measurements of Morpho-Anatomical Traits

From each pot and species, 7–10 fully expanded and healthy leaves were collected to measure the SLA and leaf thickness (L_t_) between treatments after 2 and 8 weeks of the experiment. Before collecting leaf samples at the eighth week, seedlings were first watered at runoff conditions (i.e., 40–45% VWC) to rehydrate the plants and, therefore, minimize leaf-rolling errors in the determination of leaf area. SLA was computed by dividing the leaf areas, which were measured using the leaf area meter (LI-3100, LI-COR Inc., Lincoln, United Kingdom) to the oven-dry mass of the leaves (dried at 65°C for 48 h). In this study, leaf petiole was included in the SLA measurement based on the assumption that, botanically, it is a part of a leaf because it is shed at abscission together with the leaf midrib, veins, and blades (see Pérez-Harguindeguy et al., 2013). The L_t_ was determined using a high resolution (0.01 mm) digital caliper at oven-dry weight condition. The measurement of L_t_ was consistently done in only one section (i.e., middle) of the leaves.

In this study, the variation in stomatal pore (SP) size between treatments was analyzed using the leaf epidermal impressions technique (also known as nail polish method) based on the modified procedure in Gitz and Baker (2009). From each replicate, two leaf replicas were obtained from fully expanded and healthy leaves attached to an orthotropic branch; all selected leaves had more or less similar sun exposure and internodal positions (*c.a*., 4th–6th position from the top of each plant). This was consistently done from 10:00 a.m. to 1:00 p.m. at the initial, fourth, and eighth weeks of the experiment. Samples were temporarily stored in a cold storage container until further analysis in the laboratory. Leaf replicas taken on the abaxial epidermis were observed under a compound Nikon Eclipse light microscope equipped with LAS X imaging software (Leica Microsystems Ltd., Wetzlar, Germany). A total of three images per replica were obtained. Lengths (μm) and widths (μm) of the stomatal opening (pore) of all the stomata that can be viewed at 40 × magnification on the same replica were measured using the *Image J* processing software following the procedure in some studies (Schneider et al., 2012; Li et al., 2014). For some images that were difficult to measure using the automatic function of the image processing software, we measured the lengths and width of each pore manually and averaged the values.

At second and eighth weeks, fine roots (<2 mm in diameter) were collected from randomly selected seedlings, which were the ones harvested for total soluble sugars (TSS, see section “Measurement of Total Soluble Sugar Concentration”) determination, for anatomical analysis. Before harvesting, left and right images of the belowground part of the seedlings were obtained using a flatbed scanner (Epson Perfection V37) to ensure that the samples roots were developed during stress imposition. There were a total of 12 rhizoboxes (two boxes for each treatment and species) installed in the greenhouse. Roots were washed with tap water, cut into small pieces (c.a. 5 mm long), and fixed in microcentrifuge tubes containing a 1:1 mixture of FAA-A (15 ml formaldehyde, 85 ml ethanol) and FAA-B (10 ml Glacial acetic acid and 90 ml water) for 1 month. Thereafter, samples were dehydrated using a series of alcohol concentrations (50, 65, 95, and 100% for the first, second, third, and fourth week, respectively) (Hernandez et al., 2019). Cross-sections were obtained using the freehand sectioning method (Hernandez et al., 2019) and then stained using Toluidine blue (TBO) or tolonium chloride to easily distinguish different cells and tissues in the cross-section. After mounting on a microscope slide, cross-sections were observed under a compound light microscope using only one magnification (i.e., 40×). We prepared two slides for each treatment and species. Three digital images of the cross-sections from each prepared slide were examined using the same image processing software. For each image, we measured the diameter of all the xylem vessels, which was thereafter categorized by 10 μm diameter classes. For cells/tissues in the images that were difficult to measure automatically, we obtained several measurements (length × width) across the cells/tissues and then averaged them.

The manner of taking and measuring images was consistently uniform in all the microscopic works. All microscope slides mounted with stomata and root cross sections were stored in the laboratory refrigerator at −21°C for further analysis and review.

### Measurements of Physiological Traits

The sub-stomatal CO_2_ concentration (C_i_, μmol mol^–1^) was measured using a portable photosynthesis system (LI-6400XT, LI-COR Inc., Lincoln, United Kingdom). The stomatal conductance (g_s_, mol m^–2^s^–1^) was measured using a hand-held leaf porometer (SC-1, Meter Group, Inc., United States). Measurements were conducted between 9:00 a.m. and 12:00 p.m. (KST) in the same sun-exposed, healthy, and fully expanded leaves attached to an orthotropic branch (at 5–6 nodes). C_i_ was measured with saturating light (1,500 μmol m^–2^ s^–1^), 400 μmol CO_2_ mol^–1^, 50–70% chamber relative humidity, and 26°C block temperature (i.e., average leaf temperature). Because it was difficult to determine whether the species were amphistomatous, hypostomatous, or hyperstomatous, the stomatal ratio was assumed to be 0.5 in this study.

At second and eighth periods when plants were irrigated prior to measurements of SLA and leaf thickness, measuring of the physiological traits were done 2–3 days after watering or when the VWC of the WS pots have already returned back to its initial VWC at the said periods.

Lastly, 7–10 leaves were collected for the determination of leaf relative water content (RWC) following the procedures in Jimenez et al. (2013). Leaves were immediately weighed to obtain the fresh mass (FM), petioles were immersed in water overnight, reweighed to obtain turgid mass (TM), and oven-dried at 65°C for 48 h. The RWC was then computed as (FM – DM)/(TM – DM) × 100 (Diaz-Perez et al., 1992).

### Measurement of Total Soluble Sugar Concentration

After 2 and 8 weeks, eight seedlings per treatment and species were harvested for the analysis of TSS. The first 5-cm length of stem samples from the SD mark of each seedling was cut and placed in a cold storage container until further analysis in the laboratory. Samples were immediately microwaved for 2 min to block enzymatic activities. Afterward, stem samples were debarked, oven-dried at 60°C overnight, and ground to a fine powder with a ball mill. During extraction, 50 mg of powder were suspended in 100 ml of 80% EtOH, placed in the water bath at 90°C for 10 min, and centrifuged for 10 min at 3,000 rpm. The solution was vacuum-filtered with GF/C filter paper and then adjusted the volume to 100 ml using 80% EtOH. The concentration of TSS (mg g^–1^ DW) was read at 490 nm by spectrophotometry after the phenol-sulfuric acid reaction.

### Data and Statistical Analyses

Subsampling measurements from the two seedlings planted in the middle of the pot were first averaged before statistical analysis. The effects of treatment on the growth, morpho-anatomical, physiological, and biochemical traits were tested using a one-way ANOVA for biomass between treatments. Two-way ANOVA was also used to test for significant differences in SD, height, SLA, L_t_, SP, VD, tylosis frequency (TYF), g_s_, C_i_, RWC, and TSS between treatments and among species within time points (initial, second, and eighth). We used the measured traits as dependent variables and the treatment, time, species, and their interactions as fixed factors. When treatment interaction terms were significant, means were compared using Tukey’s HSD *post hoc* test (α = 0.05). The interaction effect was visualized using the *ggpubr* R package. To identify which response mechanisms are related to drought resistance strategies, the relationships among growth, morpho-anatomical, physiological, and biochemical traits were tested using principal component analysis (PCA) for each species using the *prcomp* function. Only principal components (PCs) with eigenvalues greater than 1 were considered as important PC. We used the *factoextra* and *devtools* R packages for PCA visualization. Lastly, frequency distribution was generated to examine the frequency of xylem vessel diameter across different size classes. All the statistical analyses were performed in R statistical software (version R-3.5.1) at a significance level of α = 0.05.

## Results

### Growth Performance of the Three Species Between Treatments

**FIGURE 2:**
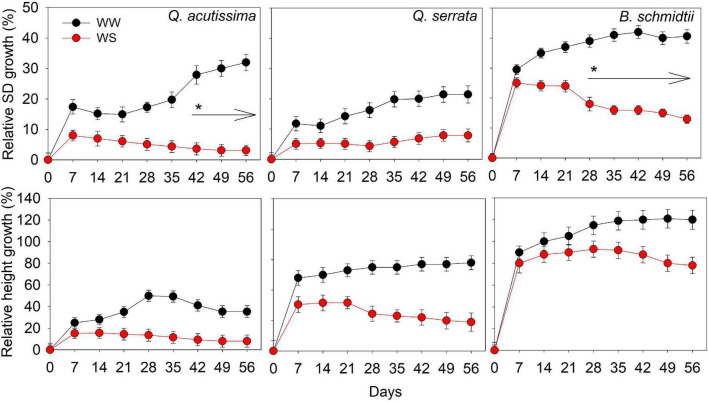
Stem diameter and height of *Quercus acutissima, Quercus serrata*, and *Betula schmidtii* in well-watered (WW) and water-stressed (WS) treatments. Vertical bars indicate the SE (*n* = 10). Different lower-case letters indicate significant differences between the treatments.

There was no significant treatment × time interaction effect detected for either SD or height in all species (Figure 2). The main treatment effect on SD was highly significant (*p* < 0.001) in all species, whereas that of time effect was only significant (*p* = 0.03) in *Q. acutissima.* The SD of *Q. acutissima* at WS-treated seedlings significantly decreased with time, such that the significant decrease started to occur at the 5th–6th weeks. WW had a significantly higher SD than WS treatment but the height was similar between treatments in all species.

**FIGURE 3:**
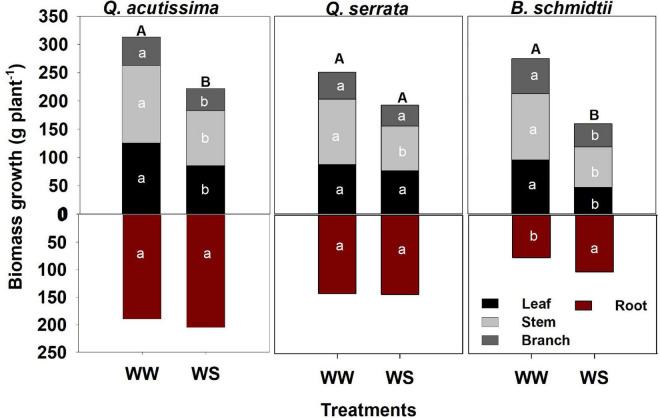
Biomass of *Quercus acutissima, Quercus serrata*, and *Betula schmidtii* in well-watered (WW) and water-stressed (WS) treatments. Different upper-case and lower-case letters, respectively, indicate significant differences in total biomass (aboveground + belowground) and biomass by plant component between treatments for each species (*n* = 10).

Total biomass was significantly higher at WW (351.78 g plant^–1^ to 503.05 g plant^–1^) than at the WS treatment (262.24 g plant^–1^ to 426.84 g plant^–1^) in *Q. acutissima* and *B. schmidtii*, whereas no difference between treatments in *Q. serrata* (Figure 3). The belowground biomass increased significantly at WS for *B. schmidtii*. Biomass allocations to leaf, stem, and branch were lower in WS compared with the WW in all species, except the leaf allocation in *Q. serrata*.

### Morpho-Anatomical Responses of the Three Species to Water Stress

No main and treatment × species interaction effects were detected for either SLA or L_t_ at the second week, but the main treatment effect on L_t_ was significant at the end of the experiment (Table 1). In the eighth week, two-way ANOVA revealed a highly significant effect (*p* < 0.001) of treatment × species interaction on SLA and L_t_. WS resulted in a smaller SLA compared with those in WW, and the degree of reduction was the highest in *Q. acutissima*, intermediate in *Q. serrata*, and the lowest in *B. schmidtii* (Table 1).

**TABLE 1:** Specific leaf area (SLA) and leaf thickness (L_t_) in well-watered (WW) and water-stressed (WS) seedlings of *Quercus acutissima*, *Quercus serrata*, and *Betula schmidtii*.

Species		SLA (cm^2^ g^–1^ DW)		L_t_ (mm)	
		After 2 weeks	After 8 weeks	After 2 weeks	After 8 weeks
*Q. acutissima*	WW	202.61 (3.52)^a^	271.50 (3.03)^a^	0.07 (0.03)^a^	0.06 (0.00)^f^
	WS	202.25 (4.16)^a^	214.15 (1.11)^b^	0.08 (0.01)^a^	0.11 (0.04)^c^
*Q. serrata*	WW	196.36 (3.04)^a^	194.22 (4.56)^c^	0.08 (0.02)^a^	0.09 (0.01)^d^
	WS	197.09 (1.18)^a^	183.20 (5.23)^d^	0.07 (0.02)^a^	0.19 (0.02)^a^
*B. schmidtii*	WW	167.59 (6.04)^a^	171.80 (4.55)^e^	0.08 (0.01)^a^	0.07 (0.01)^e^
	WS	168.87 (6.10)^a^	164.77 (2.65)^f^	0.08 (0.02)^a^	0.15 (0.01)^b^

*Different lower-case letters indicate significant differences among treatments and species. Values in parenthesis represent the SE (n = 8).*

**FIGURE 4:**
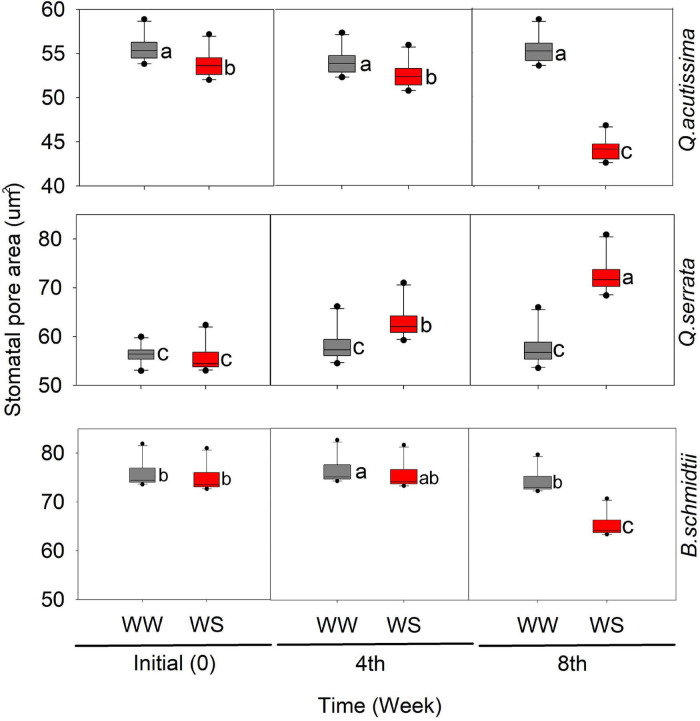
Stomatal pore area (μm) of *Quercus acutissima, Quercus serrata*, and *Betula schmidtii* in well-watered (WW) and water-stressed (WS) treatments. Different lower-case letters indicate significant differences between the two treatments (*n* = 10).

In this study, we detected significant effects of treatment, time, and their interaction on the stomatal pore area (SP) in all species (Figure 4 and Supplementary Figure 2). Time has been shown to have the greatest influence (*p* < 0.001) on SP in all species. The SP of WS-treated seedlings significantly decreased with time, while that of WW-treated ones remained similar across time periods, except in *Q. acutissima*. In *Q. serrata*, the SP at WS significantly increased compared with that at WW, and the highest change was detected at the eighth week of the experiment.

**FIGURE 5:**
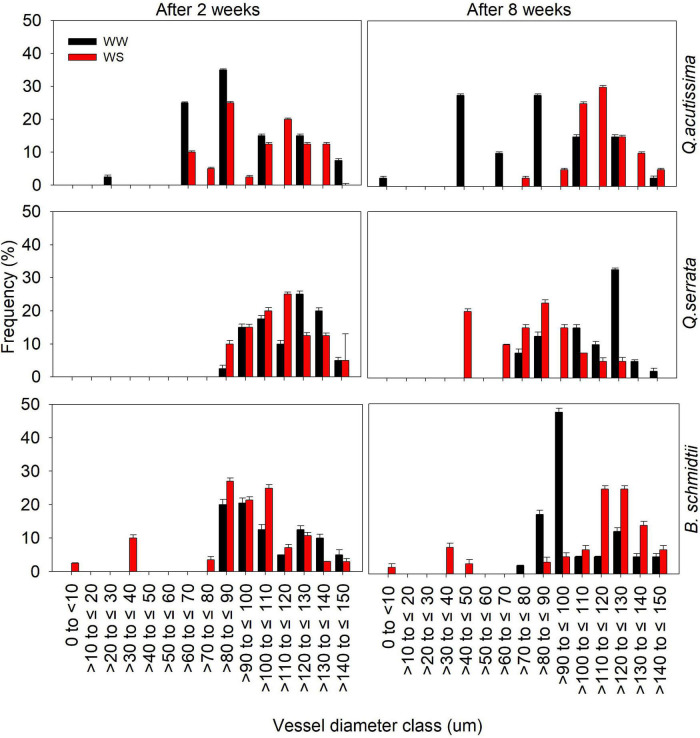
Frequency distribution of fine root xylem vessel diameter classes of *Quercus acutissima, Quercus serrata*, and *Betula schmidtii* in well-watered (WW) and water-stressed (WS) treatments. Vertical bars indicate the SE (*n* = 8).

Analysis of xylem vessels of fine root indicated differences in frequency of vessel diameter size classes between WW and WS treatments (Figure 5 and Supplementary Figure 3). After 2 weeks, the distribution of vessel diameter size classes between the treatments was generally similar, but prolonged withholding of watering resulted in contrasting responses to water stress in all species. Here, larger vessels (i.e., >100 to ≤ 130) are more frequent at WS (30–35%) than at WW (5–15%) for *Q. acutissima* and *B. schmidtii*, whereas smaller vessels (i.e., >40 to ≤ 90) are more frequent in stressed plants (20–25%) than in WW ones (10–13%) for *Q. serrata* after 8 weeks.

**FIGURE 6:**
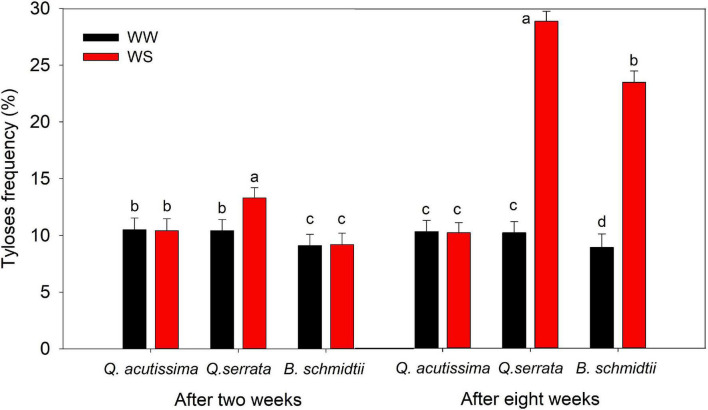
Tyloses frequency in root tracheary cells of *Quercus acutissima*, *Quercus serrata*, and *Betula schmidtii* in well-watered (WW) and water-stressed (WS) treatments. Vertical bars indicate the SE (*n* = 8). Different lower-case letters indicate significant differences between the two treatments across species.

In this study, the frequency of tyloses or outgrowths in root tracheary cells also varied significantly by treatment (*p* < 0.001), species (*p* < 0.001), and their interaction (*p* = 0.04), particularly after 8 weeks of the experiment (Figure 6 and Supplementary Figure 4). Tylosis was more frequent in WS than at WW for *Q. serrata* and *B. schmidtii* at eighth week, whereas did not vary in the case *Q. acutissima*. Across species, *Q. serrata* had the highest TYF, intermediate in *B. schmidtii*, and the lowest in *Q. acutissima*.

### Physiological Response of the Three Species to Water Stress

**FIGURE 7:**
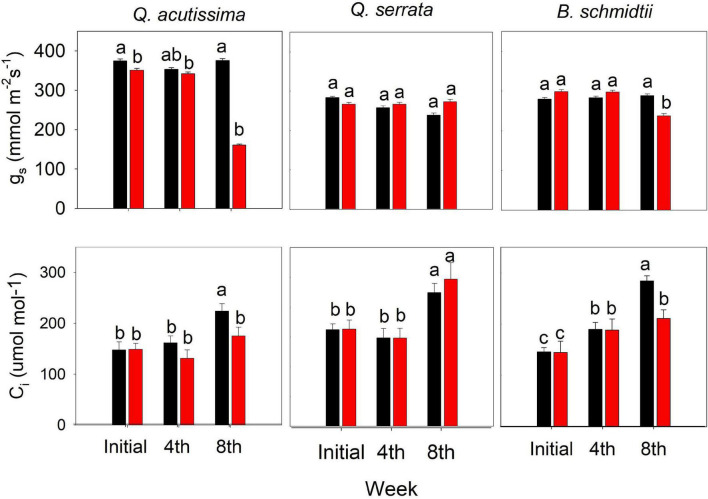
Sub-stomatal CO_2_ concentration (C_i_) and stomatal conductance (g_s_) of *Quercus acutissima, Quercus serrata*, *and Betula schmidtii* in WW (well-watered) and WS (water-stressed) treatments. Vertical bars indicate the SE (*n* = 10). Different lower-case letters indicate significant differences between treatments across time.

Some of the physiological traits measured in this study varied significantly by treatment, time, and their interactions (Figure 7). The treatment × time interaction has been shown to have the greatest influence on the stomatal conductance (g_s_) for *Q. acutissima* (*p* < 0.001) and *B. schmidtii* (*p* = 0.003). Only at the eighth week was g_s_ significantly decreased and C_i_ increased for *Q. acutissima* and *B. schmidtii*, whereas no change was detected in *Q. serrata* at WS treatment.

We did not detect treatment, species, and interaction effects on leaf RWC during the first 2 weeks of the experiment, but highly significant interaction effects (*p* < 0.001) were detected after 8 weeks (Table 2). The leaf RWC was generally significantly lower at WS-treated seedlings (i.e., 72.48% for *Q. serrata* > 31.50% for *Q. acutissima* = 26.19% for *B. schmidtii*) than that at the WW-treated ones (i.e., 89.05% for *Q. serrata* = 86.98% for *B. schmidtii* = 76.79% for *Q. acutissima*). *Q. serrata* maintained the highest RWC in both treatments among the three species at the end of the experiment.

**TABLE 2:** Leaf relative water content (RWC) of *Quercus acutissima*, *Quercus serrata*, and *Betula schmidtii* in well-watered (WW) and water-stressed (WS) treatments.

Species	Treatments	RWC (%)	
		After 2 weeks	After 8 weeks
*Q. acutissima*	WW	75.79 (0.49)^a^	76.79 (1.05)^a^
	WS	75.95 (1.25)^a^	31.50 (0.90)^b^
*Q. serrata*	WW	90.05 (1.10)^a^	89.05 (1.90)^a^
	WS	89.92 (1.28)^a^	72.48 (1.02)^a^
*B. schmidtii*	WW	85.79 (1.49)^a^	86.98 (0.92)^a^
	WS	85.98 (1.25)^a^	26.19 (1.02)^b^

*Different lower-case letters indicate significant differences among treatments and species. Values in parenthesis represent the SE (n = 8).*

### Variation in Total Soluble Sugar Concentration Between Treatments

**FIGURE 8:**
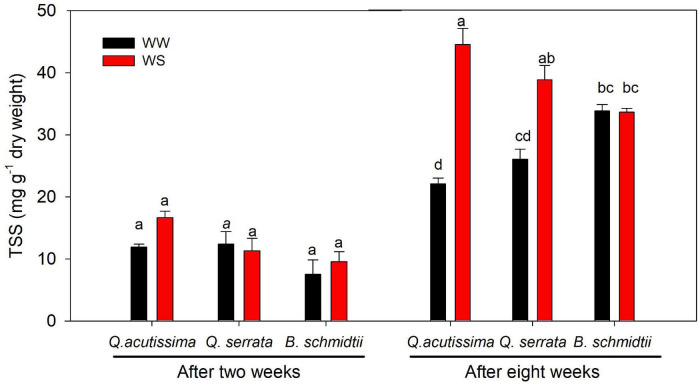
Total soluble sugars (TSS) of *Quercus acutissima, Quercus serrata*, and *Betula schmidtii* in well-watered (WW) and water-stressed (WS) treatments. Vertical bars indicate the SE (*n* = 8). Different lower-case letters indicate significant differences between the two treatments across species.

After 2 weeks, the treatment, species, and their interaction showed no significant effect on the concentration of total soluble sugars (TSS), but a highly significant effect (*p* = 0.005) of the interaction was detected after 8 weeks (Figure 8). WS treatment resulted in significantly higher TSS concentration than WW, particularly in two *Quercus* species. Here the TSS concentration was found the highest at WS-treated seedlings of *Q. acutissima*, intermediate in *Q. serrata*, and the lowest in *B. schmidtii*.

### Principal Component Biplot Analysis

**FIGURE 9:**
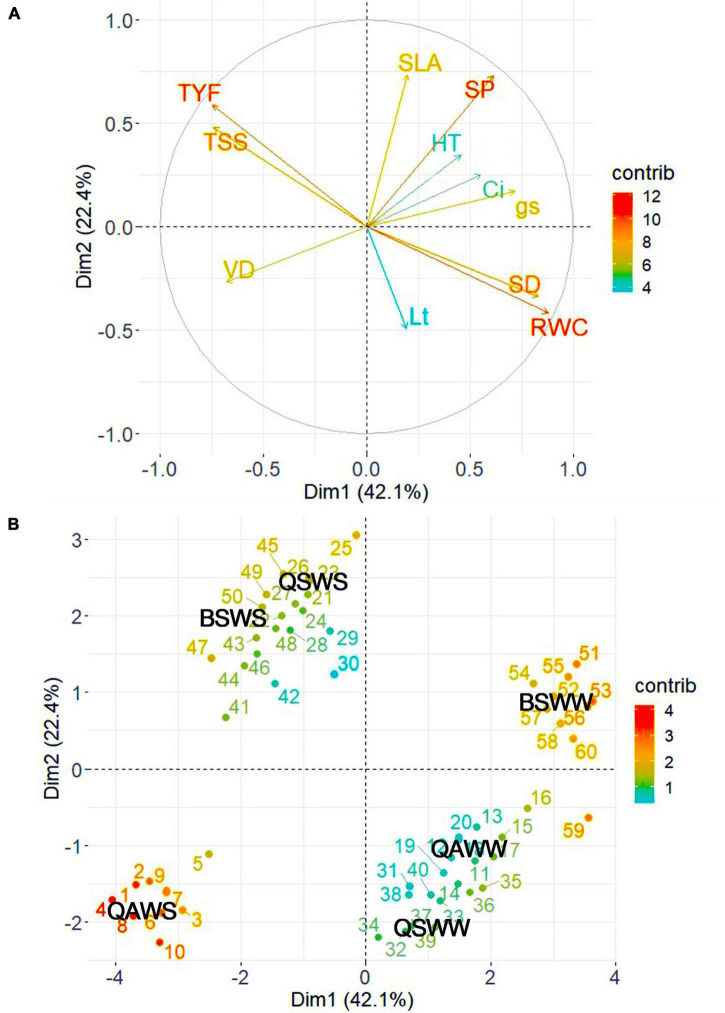
Principal component loading plot of the variables **(A)** and individuals **(B)** observed from Q. *acutissima* (QS), *Q. serrata* (QS), and *B. schmidtii* (BS) in well-watered (WW) and water-stressed (WS) at eighth week of the experiment. SD, stem diameter, SLA, specific leaf area, L_t_, leaf thickness, SP, stomatal pore area, VD, vessel diameter, TYF, tylosis frequency, g_s_, stomatal conductance, C_i_, intercellular CO_2_ concentration, RWC, relative water content, TSS, total soluble sugar. The “contrib” denotes contribution of the variables/individuals to dimensions.

To determine the contributions of each variable measured in the WW and WS-treated seedlings of the three species, we performed PCA using the growth, morpho-anatomical, physiological, and biochemical traits (Figure 9 and Supplementary Tables 1, 2). The first two components accounted for 64.50% of the variation in the data set. Specifically, PC 1 accounted for 42.10% of the variation and was highly related to RWC, SD, g_s_, TYF, TSS, and VD. PC 2 accounted for 22.40% of the variation and was highly related to SLA and SP. The RWC and SD were positively correlated with control or WW-treated seedlings of *Q. acutissima* and *B. schmidtii*. TYF, TSS, and VD were strongly associated with the WS-treated seedlings of *Q. acutissima*, whereas SLA and SP were positively correlated with WS-treated seedlings of *Q. serrata* and B. *schmidtii.*

## Discussion

### Tylosis Frequency, Soluble Sugar Accumulation, and Vessel Diameter Are Strongly Associated With Water-Stressed *Quercus acutissima*

ANOVA and PCA biplot showed the growth, morpho-anatomical, physiological, and biochemical traits that are related to water stress resistance in *Q. acutissima, Q. serrata*, and *B. schmidtii*. In this study, TYF, TSS, and VD were strongly associated with water-stressed seedlings of *Q. acutissima*, suggesting that water stress resistance strategies of the species were mainly controlled by anatomical (tylosis and xylem vessel) and biochemical traits (soluble sugar). Because most of the physiological traits declined in *Q. acutissima* at the end of experiment, the species may have increased the frequency of larger xylem vessels in fine root to structurally adapt to large changes in pressure and survive amid progressive water stress. Several studies reported that larger xylem vessels resulted in higher hydraulic conductivity but are highly correlated with higher xylem vulnerability to embolism (Lovisolo and Schubert, 1998; Quintana-Pulido et al., 2018). Even in a long-term drought experiment, results illustrated more collapsed xylem vessels in the root of poplars under the drought treatment compared with the WW one (Zhou et al., 2020). However, we did not detect any signs of embolism in xylem vessels of *Q. acutissima*, and that can be attributed to similar TYF between WW and WS at the end of the experiment. This is because under the drought condition rapid tylosis formation in vessels can cause vessel occlusions that can trigger a dramatic decrease in sap flow (McElrone et al., 2010) and large pressure differences (Augé, 2001). Our result is consistent with the study of Murmanis (1975), who showed no negative effect of tyloses despite the significant accumulation under abiotic stress in *Quercus rubra* L. Several studies showed that leaf scorching, shedding, and wilting are common symptoms associated with vascular dysfunctions because xylem vessels were occluded by an embolism (Tyree and Zimmermann, 2002; Hochberg et al., 2017).

In this study, a significantly higher amount of TSS was observed in WS-treated seedlings than WW-treated ones in *Q. acutissima*. Consistent with the results reported in some studies, it was shown that a decline in leaf water potential in WS plants was counteracted by a significant increase in sugar concentration by promoting turgor maintenance (Silva and Arrabaça, 2004; Bandurska et al., 2009; Krasensk and Jonak, 2012; Falchi et al., 2020). Advanced studies on metabolic engineering to increase the plant tolerance to environmental stresses have also shown that increased accumulation of TSS was related to drought and cold tolerances (Rathinasabapathi, 2000). Soluble sugars are involved in the regulation of water transport, embolism formation, and hydraulic conductivity (Tomasella et al., 2020). Hence, the increased frequency of larger vessels could further explain the observed higher TSS in *Q. acutissima*. This is because xylem vessels serve as the main storage sites of non-structural carbohydrates (NSC) and fulfill important functions, such as osmoregulation and direct exchange of ions, water, and soluble sugars with xylem sap in woody plants (Secchi and Zwieniecki, 2011; Morris et al., 2018). A review by Tomasella et al. (2020) found that embolism formation is positively associated with the depletion of NSC in stem, and such depletion is attributed to the consumption of soluble sugars during post-stress recovery. Therefore, there was really no serious xylem cavitation that occurred in *Q. acutissima* because we still observed similar TFY between WW and WS and a higher amount of TSS even at the end of the experiment. This further suggests that the intensity and duration of stress we imposed may have not yet enough to cause carbon starvation, embolism, and NSC depletion. Thus, the WS seedlings of *Q. acutissima* may still tolerate prolonged water stress by keeping their TSS high and available until the recovery phase for vigor and survival.

*Q. acutissima* may have benefited from the accumulation of TSS by allowing a gradual decline in RWC and g_s_ to keep growing and surviving as the stress progressed. This decline in the above traits is further supported by the observed significant decrease in SP, which was strongly and positively correlated with most of the physiological traits. A study by Rathinasabapathi (2000) explained also that sugar concentration in plants can modulate several physiological processes, thereby providing a certain level of tolerance against abiotic stresses, and this, in part, confirms the observed negative correlation between TSS and all the physiological traits measured in *Q. acutissima* based on the PCA plot. A decrease in photosynthetic activity due to water stress is normally experienced by plants due to the decline in CO_2_ conductance *via* increased stomata closure (Singh and Thakur, 2018). Studies have shown that continued stomata opening increases osmotic stress that can lead to severe damage to photosynthetic machinery and excessive water loss *via* evapotranspiration in plants under increasing water stress duration (Zare et al., 2011; Bhargava and Sawant, 2013). Here, the significant decline in SP of *Q. acutissima* under water stress indicates stomatal closure, which may have avoided excessive water loss *via* evapotranspiration and damage to the physiological machinery of the seedlings.

### Specific Leaf Area and Stomatal Pore Area Are Strongly Associated With Water-Stressed *Quercus serrata* and *Betulas chmidtii*

Results revealed that SLA and SP were strongly associated with WS-treated seedlings of *Q. serrata* and *B. schmidtii.* Here, the effect of the treatment × species interaction was found to have the greatest influence on SLA at the eighth week of the experiment. Their SLA significantly decreased when exposed to water stress, and the magnitude of the decrease varied considerably across species. This result is consistent with some studies indicating that drought-exposed plants have smaller SLA (Cutler et al., 1977; Niinemets, 2001; Liu et al., 2019). Our finding can be attributed to the observed variations in relative height growth across species. Here, *B. schmidtii* and *Q. serrata* had a significantly higher height growth than *Q. acutissima* regardless of treatment and time period. The tallest seedlings of the *B. schmidtii* and *Q. serrata* may have resulted in a substantial decrease in leaf area to keep surviving amid decreasing soil moisture, by minimizing the use of water for photosynthesis and water loss *via* evapotranspiration. In 16 temperate tree species, Wang et al. (2019) found that the smaller the leaves are, the faster the leaf water losses, which supports the order of reduction we found for RWC (i.e., *B. schmidtii* > *Q. acutissima* > *Q. serrata*). Further, light availability may have been higher at the top of the tall seedlings of *B. schmidtii* and *Q. serrata*, so there was no need to expand their leaf area for light resource acquisition especially that they were already under water-deficient condition. Contrarily, shorter seedlings of *Q. acutissima* may have been exposed to a lower light intensity due to shading by the other plants, thereby expanding its leaf area for light acquisition. Result agrees with the experiment of Błasiak et al. (2021) who also found the smallest SLA in the upper part of the plant compared with the other parts, and the findings were attributed to light exposure.

Regarding hydraulic traits, the SP of *B. schmidtii* decreased and increased in the case of *Q. serrata* as the duration of water stress increased. Plants regulate stomata by controlling the pore opening and closure to maintain their marginal hydration at the expense of carbon gain (Zenes et al., 2020). The observed smaller pore area in WS-treated leaves of *B. schmidtii* may be interpreted as a drought response mechanism of the species to keep hydrated amid decreasing soil moisture. Wang et al. (2019) discovered that leaf water loss rate (*k*) was significantly negatively correlated with the stomatal size, which further explains the highest degree of reduction of RWC in *B. schmidtii*. WS-treated seedlings of *B. schmidtii* may have closed its stomata to control the increasing water loss under the progressive water stress. During the dry season, some trees maintain very low transpiration and photosynthesis rate as a consequence of prolonged or rapid stomatal closure or decline in stomatal conductance (Fanourakis et al., 2014). If the stomata of *B. schmidtii* remained open even at high-level water stress (i.e., eighth week), transpiration rate could have been very high, which could have led to blocking of xylem conduits by air bubbles (embolism) as the soil moisture deficits increased. This explains the observed positive correlation between SP and the physiological traits in *B. schmidtii* based on the PCA plot. The result found in *B. schmidtii* is consistent with results reported in Mano et al. (2020), who reported a significant reduction in the stomata pore area of *Betula nigra* L. in response to water stress *via* stomatal development plasticity. The author further noted that despite such a significant decrease, there was only a minor to no effect on the stomatal conductance of the species. Similarly, a study by Zenes et al. (2020) reported that water stress resulted in increased stomatal sensitivity (i.e., conservative behavior or stomatal closure). Further, the molecular basis for stomatal plasticity to water stress has already been reported (Viger et al., 2016; Bertolino et al., 2019). A review also concluded that recent studies are already showing the potential of modification of stomata-related traits (e.g., number and size) to be an effective tool for inducing plant WUE and drought tolerance (Bertolino et al., 2019).

Moreover, smaller vessels of *B. schmidtii* were more frequent at WW than WS, but as the stress progressed, the frequency of larger VD of fine root increased at WS treatment. This can also be linked to the continued decline in SP, which may have stimulated the root cells to be more efficient at water absorption and uptake *via* enlarged vessels. McElrone et al. (2004) found that mean vessel diameter was significantly greater in deep roots than shallow roots, and this finding can be supported by a significant increase in root biomass of *B. schmidtii* at WS treatment. The significant decrease in SP of the species can also be interpreted as a mechanism to control the over-accumulation of tylosis, which can cause embolism in roots. This seems to agree with the observed similar TSS between WW and WS, suggesting that the soluble sugar may have been already started to be consumed in response to carbon starvation caused by stomatal closure and tylosis build-up at the end of the experiment.

In the case of *Q. serrata*, the increase in stomatal pore size may indicate high stomatal plasticity to progressive water stress to adapt to the prevailing soil condition. Leaves with larger stomatal pores tend to have higher stomatal conductance and photosynthetic rates, but may also be prone to mesophyll damage and xylem embolism during extreme water stress (Martin-StPaul et al., 2017). In contrast to our result, Fotelli et al. (2000) reported a remarkable reduction in stomatal aperture of some drought-treated *Quercus* species, but the species still yielded a very low water potential despite such stomatal aperture adjustment during drought. However, the study by Ogasa et al. (2013) seems to agree with our observation, such that the continued stomatal opening was observed in ring-porous *Betula* species despite xylem embolism during water stress experiment (Ogasa et al., 2013). Such a sustained stomatal opening was considered as a potential mechanism for attaining sustainable gas exchange, higher xylem-specific hydraulic conductivity, and optimizing CO_2_ uptake while minimizing water loss despite xylem cavitation or dysfunction or gradual leaf shedding (Ogasa et al., 2013). The fluxes of CO_2_ and water in leaves are influenced by soil moisture content, stomatal pore aperture, guard cell turgor pressure, and hydraulic conductivity. In this study, despite the continued opening of the stomatal pore of the species at WS treatment, we did not observe a serious reduction in hydraulic conductivity of *Q. serrata* because its RWC was still within the reported normal value (i.e., 72.48%) (Kalaji and Nalborczyk, 1991; Reddy et al., 2004; Singh and Reddy, 2011). This may have enabled the species to fully open its stomatal aperture even at high-level stress, leading to increased C_i_ and maintained physiological functions, but with minimal water loss *via* leaf transpiration, as shown by its high RWC. The higher C_i_ observed in *Q. serrata* under WS treatment particularly at the eighth week is, therefore, expected because of its maintained higher RWC and larger stomatal pore size even after 8 weeks of the experiment. As the stress was progressing, a small amount of water may have gradually and regularly been delivered from the roots to leaves of *Q. serrata*, resulting in turgid and opened stomata. Moreover, opened stomata and maintained high RWC amid increasing duration of stress imposition can also be attributed to the dense layers of glandular and non-glandular trichomes in the leaves of *Q. serrata* (Supplementary Figure 5). The role of trichomes in water stress tolerance and water economy *via* improvement of water use efficiency and reduction of transpiration rate has already been described in many studies. For example, plants with a high density of trichomes resulted in a higher drought tolerance compared with those plants with low trichome density (Ewas et al., 2016). Especially when stomata are open, trichomes can provide shade on the leaf surface to reduce water *via* evapotranspiration (De Micco and Aronne, 2012; Hernandez et al., 2019).

In *Q. serrata*, we also found a higher TSS concentration at WS than WW at the eighth week. Although TSS accumulation is not associated with WS-treated seedlings of the species based on the PCA loading plot, this may have helped the species survive water stress, maintained the g_s_ and increased SD growth or remain alive under increasing water stress levels. However, we can also say that, in the longer duration of water stress, TSS accumulation may also be associated with water stress resistance in *Q. serrata.* This is because several studies reported that the presence of a high concentration of TSS maintained the leaf water content and osmotic adjustment of plants grown under water stress (Xu et al., 2007; Dien et al., 2019), and this can further explain the observed similar RWC between WW and WS in *Q. serrata.*

Further, in contrast to *Q. acutissima*, i.e., lower TYF with larger VD, *Q. serrata* had a higher TYF but with smaller VD. Tyloses can aid the formation of stronger heartwood cells by blocking larger vessels with outgrowths and slowing abiotic damage to plant cells (Esau, 1965; Thomas et al., 2015). A study by Feng et al. (2013) stated that tyloses formation indicated ecophysiological responses to constant environmental stimuli in Permian conifer stem. Effects of water stress can be triggered by an increasing number of vessel blockages of tyloses, which can significantly affect the uptake and utilization of carbon under the prolonged duration of stress (Brodribb and Holbrook, 2006; Ingel et al., 2020). Overproduction of tyloses, thus, can result in a serious reduction in hydraulic conductivity within the xylem (Tyree and Zimmermann, 2002; McElrone et al., 2010). However, overproduction of tylosis seemed to be not feasible for *Q. serrata* because of the increased frequency of the smaller diameter of xylem vessels in WS-treated seedlings. Studies have shown that small diameter is associated with high tolerance to xylem embolism (Cochard, 2002; Smith et al., 2013). This adjustment in the hydraulic architecture seemed to be associated with the continued stomatal opening at prolonged water stress, thereby allowing the species to tolerate the adverse effect of water stress *via* osmotic adjustment.

Overall, water-stressed plants from all species responded positively to water stress with increasing the experimental duration and stress intensity, and that is largely explained by morpho-anatomical traits and soluble sugar concentration. Results suggest that these traits help them to avoid, escape, and/or tolerate drought conditions. The present study should enhance our understanding of drought-induced tree growth and short-term tree-seedling responses to drought. Further, our findings are also relevant to providing economic and ecological insights into species-site suitability assessment for tree planting programs, particularly in dry areas, and survival and water-resource competition strategies across the studied species when planted under water stress sharing the same habitat.

## Data Availability Statement

The datasets presented in this study can be found in online repositories. The names of the repository/repositories and accession number(s) can be found below: Harvard Dataverse: https://doi.org/10.7910/DVN/4EDS3B.

## Author Contributions

JOH, BBP, and JYA initiated the research project. JOH and BBP defined the research question, designed the experiments, and finalized the manuscript. JOH collected, analyzed the data, and wrote the manuscript. BBP, JYA, MSC, JPC, and SKO supervised, commented and revised the manuscript. BBP acquired and managed the funding. All authors contributed to the article and approved the submitted version.

## Conflict of Interest

The authors declare that the research was conducted in the absence of any commercial or financial relationships that could be construed as a potential conflict of interest.

## Publisher’s Note

All claims expressed in this article are solely those of the authors and do not necessarily represent those of their affiliated organizations, or those of the publisher, the editors and the reviewers. Any product that may be evaluated in this article, or claim that may be made by its manufacturer, is not guaranteed or endorsed by the publisher.
