# Supplementary material for: Morpho-Anatomical Traits and Soluble Sugar Concentration Largely Explain the Responses of Three Deciduous Tree Species to Progressive Water Stress
Source: Front Plant Sci. 2021 Dec 7;12:738301. doi: 10.3389/fpls.2021.738301 (PMC8688917; doi:10.3389/fpls.2021.738301)
Supplement: Supplementary file 1 [file Data_Sheet_1.doc]

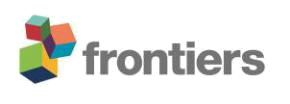


***Supplementary Material***


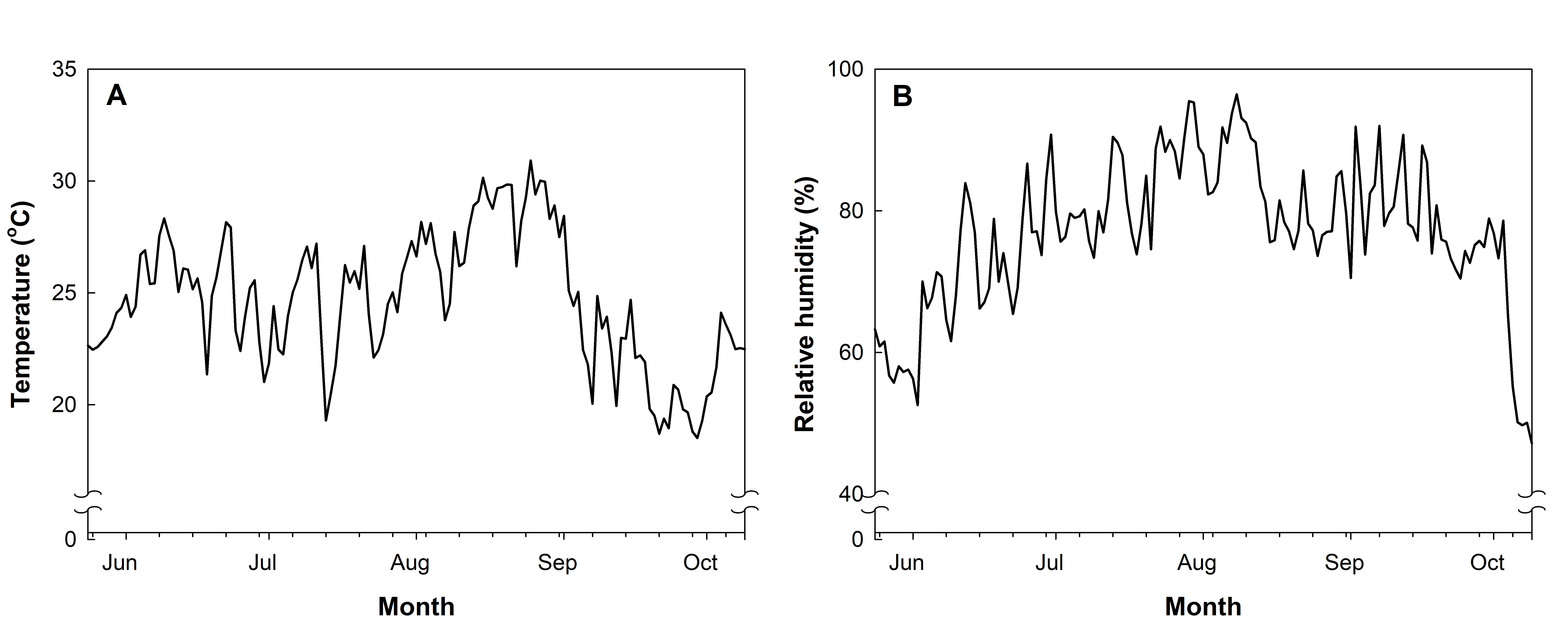


**Supplementary Figure 1**. Mean daily temperature (A) and relative humidity (B) in the greenhouse during the study period (June-October 2020).

***
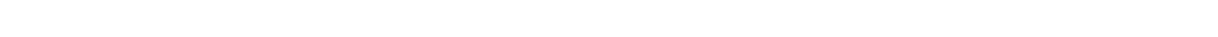
***

*Quercus acutissima* *Quercus serrata* *Betula schmidtii*

*
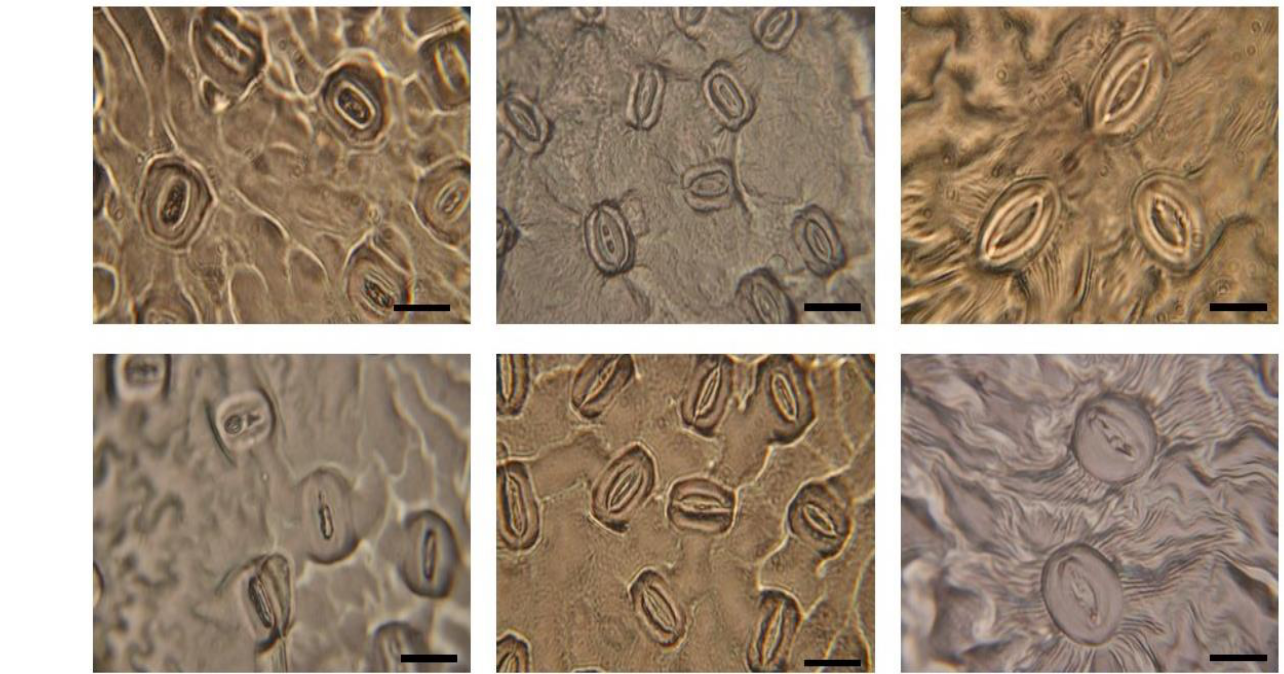
*

**WW**

**decreased** **increased** **_decreased_**

**WS**

**Supplementary Figure 2.** Representative stomata samples of *Quercus acutissima*, *Quercus serrata,* and *Betula schmidtii* in WW (well-watered) and WS (water-stressed) treatments after eight weeks of the experiment. Scale bar represents 5.02 µm.

Supplementary Material


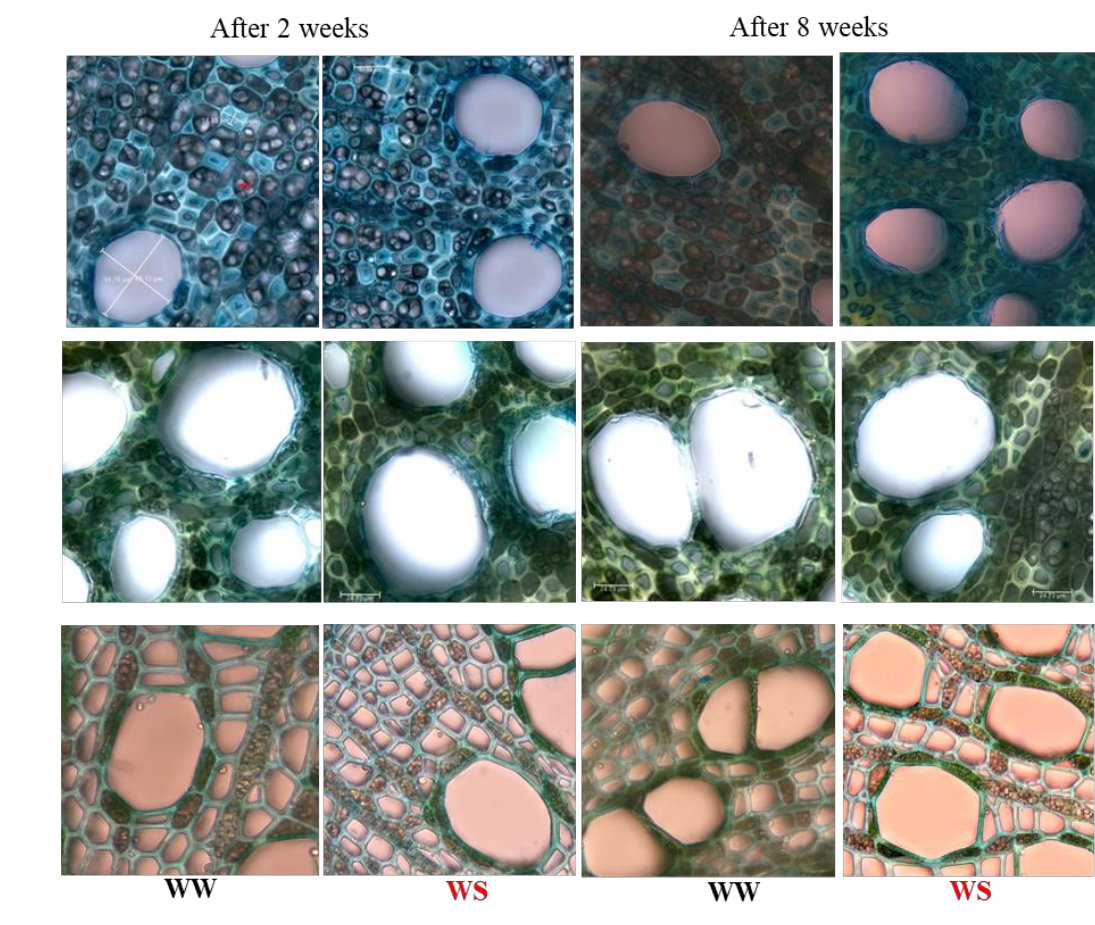


| *Betula schmidtii Quercus serrata Quercus acutissima* |
| --- |

**Supplementary Figure 3.** Representative xylem vessels of fine roots of *Quercus acutissima*, *Quercus serrata,* and *Betula schmidtii* in WW (well-watered) and WS (water-stressed) treatments.

2

*Quercus acutissima* *Quercus serrata* *Betula schmidtii*

*
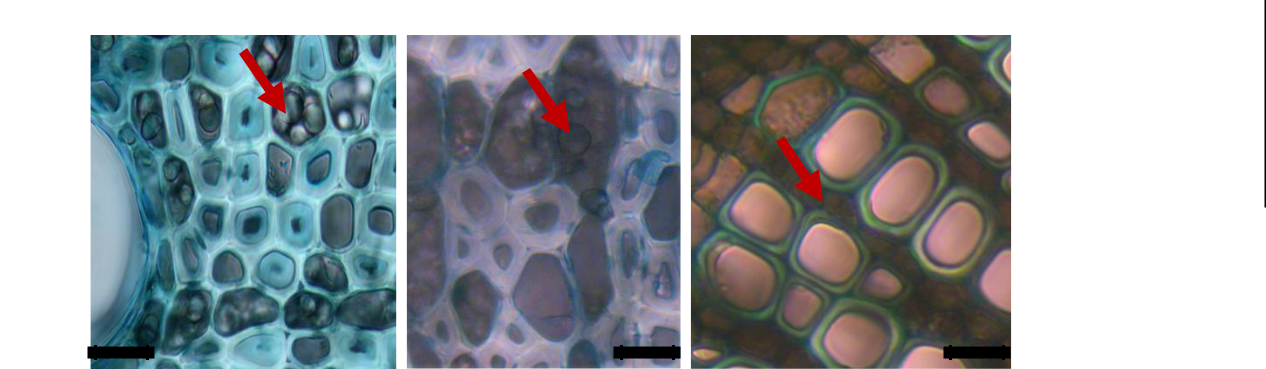
*

**WW**

**
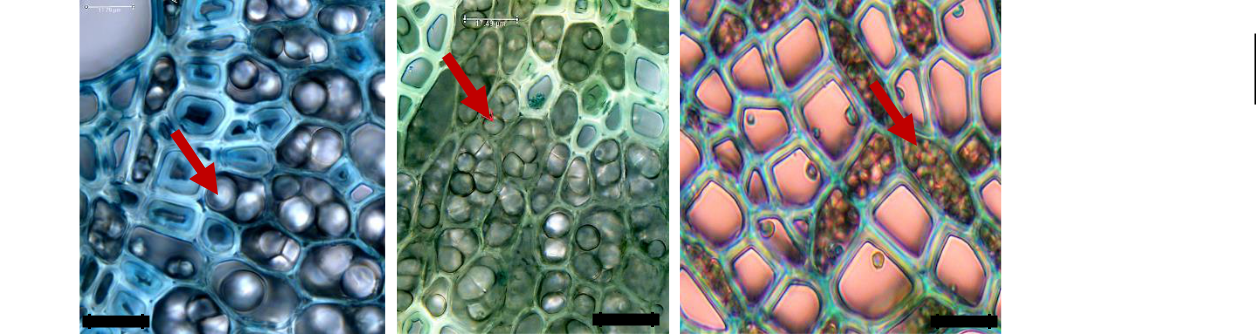
**

**WS**

**Supplementary Figure 4.** Representative tylosis in fine root tracheary cells of *Quercus acutissima*, *Quercus serrata,* and *Betula schmidtii* subjected to WW (well-watered) and WS (water-stressed) treatments after eight weeks. Scale bar represents 17.79 µm. Red arrow indicates tylosis.

3

Supplementary Material


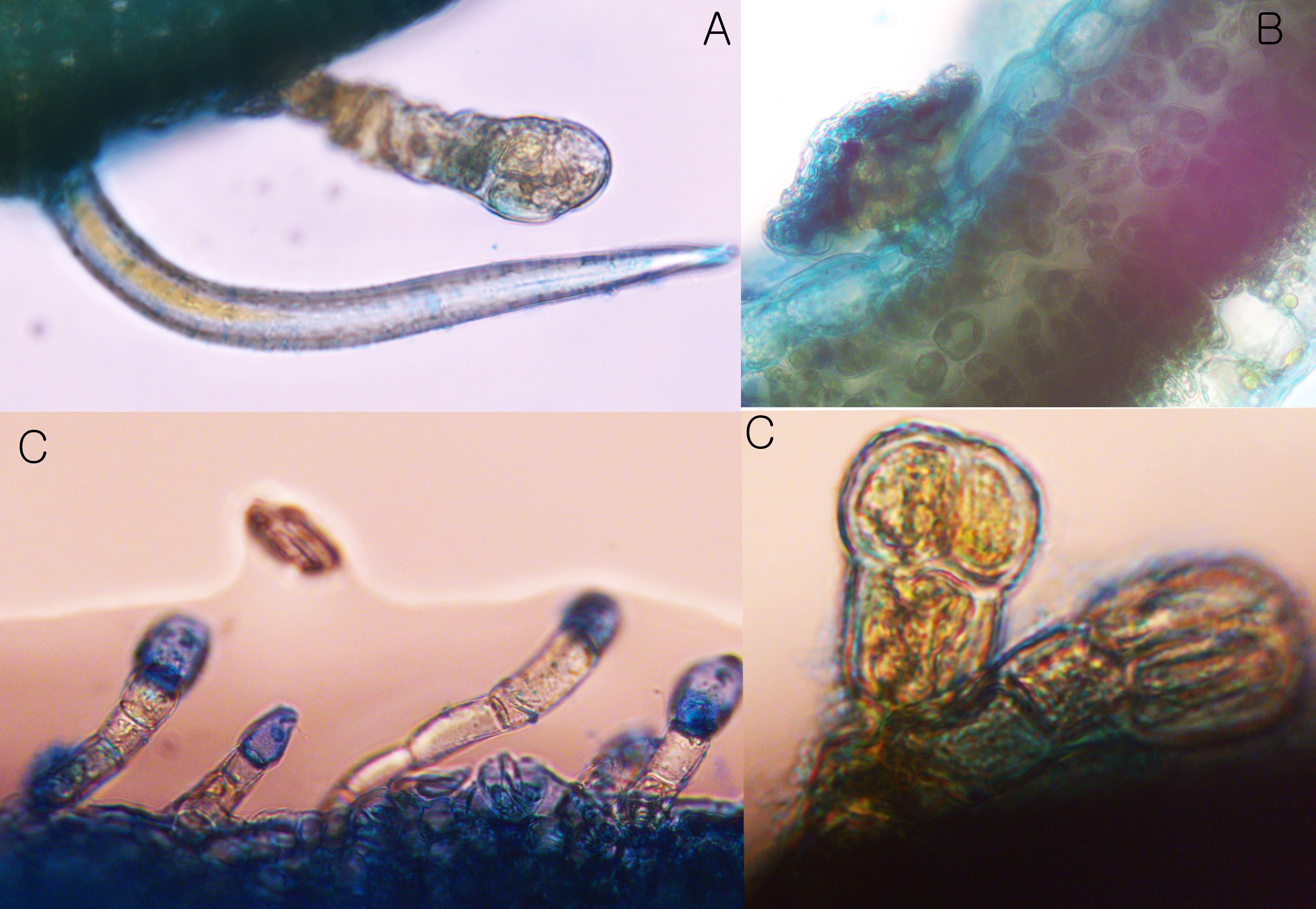


**Supplementary Figure 5.** Trichomes on the abaxial leaf surface of *Quercus serrata* showing (A) glandular capitate with long stalk and non-glandular cylindrical, (B) glandular peltate (C) glandular conical, and (D) glandular capitate with short stalk.

**Supplementary Table 1.** Eigenvalues of the twelve components/dimensions of the principal component analysis loading plot.

|  |  |  | Cumulative variance |
| --- | --- | --- | --- |
| Components | Eigenvalue | Variance percent | percent |
| Dim.1 | 5.04 | 42.07 | 42.07 |
| Dim.2 | 2.68 | 22.35 | 64.43 |
| Dim.3 | 1.66 | 13.91 | 78.34 |
| Dim.4 | 0.81 | 6.79 | 85.13 |
| Dim.5 | 0.60 | 5.03 | 90.17 |
| Dim.6 | 0.47 | 3.98 | 94.15 |
| Dim.7 | 0.38 | 3.18 | 97.34 |
| Dim.8 | 0.13 | 1.15 | 98.49 |
| Dim.9 | 0.08 | 0.73 | 99.22 |
| Dim.10 | 0.05 | 0.49 | 99.72 |
| Dim.11 | 0.02 | 0.20 | 99.92 |
| Dim.12 | 0.00 | 0.07 | 100 |

**Supplementary Table 2.** Contribution of the variables to the first two components/dimensions of the principal component analysis loading plot.

|  | Dim.1 |  | Dim.2 |
| --- | --- | --- | --- |
| SD |  | 0.831 | -0.339 |
| HT |  | 0.454 | 0.347 |
| SLA |  | 0.197 | 0.735 |
| Lt |  | 0.191 | -0.492 |
| SP |  | 0.614 | 0.731 |
| VD |  | -0.676 | -0.265 |
| TYF |  | -0.746 | 0.588 |
| A |  | 0.740 | 0.467 |
| gs |  | 0.718 | 0.171 |
| Ci |  | 0.552 | 0.250 |
| RWC |  | 0.879 | -0.418 |
| TSS |  | -0.739 | 0.481 |

4
